# Disentangling the intricate atomic short-range order and electronic properties in amorphous transition metal oxides

**DOI:** 10.1038/s41598-017-01151-2

**Published:** 2017-05-17

**Authors:** C. A. Triana, C. Moyses Araujo, R. Ahuja, G. A. Niklasson, T. Edvinsson

**Affiliations:** 1 Division of Solid State Physics, Department of Engineering Sciences, The Ångström Laboratory, Uppsala University, P.O. Box 534, SE-751 21 Uppsala, Sweden; 2 Materials Theory Division, Department of Physics and Astronomy, Uppsala University, P.O. Box 516, SE-75120 Uppsala, Sweden

## Abstract

Solid state materials with crystalline order have been well-known and characterized for almost a century while the description of disordered materials still bears significant challenges. Among these are the atomic short-range order and electronic properties of amorphous transition metal oxides [aTMOs], that have emerged as novel multifunctional materials due to their optical switching properties and high-capacity to intercalate alkali metal ions at low voltages. For decades, research on aTMOs has dealt with technological optimization. However, it remains challenging to unveil their intricate atomic short-range order. Currently, no systematic and broadly applicable methods exist to assess atomic-size structure, and since electronic localization is structure-dependent, still there are not well-established optical and electronic mechanisms for modelling the properties of aTMOs. We present state-of-the-art systematic procedures involving theory and experiment in a self-consistent computational framework to unveil the atomic short-range order and its role for the electronic properties. The scheme is applied to amorphous tungsten trioxide aWO_3_, which is the most studied electrochromic aTMO in spite of its unidentified atomic-size structure. Our approach provides a one-to-one matching of experimental data and corresponding model structure from which electronic properties can be directly calculated in agreement with the electronic transitions observed in the XANES spectra.

## Introduction

Amorphous transition metal oxides [aTMOs] are key components in optoelectronics, sensors, photoelectrochemical conversion, energy/data storage and emerging water splitting applications, where extensive research on their technological optimization has been performed^1^. However, unveiling their intricate atomic short-range order and the underlying electronic properties is challenging because no systematic and well-established methods exist to achieve such tasks. Structural analysis of amorphous materials by X-ray/neutron pair distribution function [PDF], requires precise data corrections and complex data analysis depending on the diffraction geometry. For disordered materials exhibiting large multiple-scattering phenomena such as complex oxides and perovskite materials it has been shown that the PDF is unreliable to recover the statistics of many-body correlations in the nearest coordination shells, thus, underestimating the distribution of atomic coordination^2^. Standard X-ray-absorption spectroscopy [XAS-fitting], is applied to small atomic clusters without periodic boundary conditions to optimize structural parameters; Debye-Waller factors *σ* ^2^, and relative weights of scattering-paths^3^. However, XAS-fitting merely provides average values of structural parameters, but not the full 3D-structure of the system. The absence of short-range order leads to a distribution of interatomic distances, bond-angles and atomic coordinations^4^, which cannot be well-resolved by standard XAS-fitting. As stated by Anderson, Mott and Cohen *et al*.^5–7^, disorder induces electron localization near the energy-gap edges, yielding band-tails of occupied and unoccupied localized states extended into the mobility gap. However, due to the difficulty in determining local disorder, there is still no well-established electronic localization mechanisms for aTMOs. Unveiling atomic short-range order is thus crucial to understand optical and electronic processes in aTMOs and would also be of vital importance in nanoparticle systems where the high-surface-to-bulk ratio results in lower crystallinity arising from structural reconstruction and surface disorder^8, 9^.

Here we present a state-of-the-art systematic computational procedure based on XAS experiments to assess the atomic short-range order and electronic properties of aTMOs. The scheme is applied to *a*WO_3_, the leading electrochromic aTMO for application in energy-efficient “smart windows”. This technology offers unique optical switching functionalities through cyclic inter/deintercalation of alkali metal ions [Li^+^]^10, 11^. There are so far no comprehensive studies assessing the atomic short-range order of *a*WO_3_, in spite that such knowledge can aid to tune and enhance functionalities. Although molecular dynamics [MD] has been used to assess atomic short-range order in *a*WO_3_. However, several structural artifacts have been reported and simulated structures were also unable to fit the experimental data^12, 13^. In the present scheme, the atomic short-range order of *a*WO_3_ is instead extracted from reverse Monte Carlo [RMC] simulation of the experimental extended X-ray-absorption fine structure [EXAFS] spectra. This RMC-EXAFS approach overcomes the main drawbacks of standard EXAFS-fitting since it provides optimized 3D-structures and related parameters. The inclusion of multiple-scattering terms yields an explicit treatment of three-body correlations^2, 3^. The effects of static disorder due to the fluctuation of interatomic distances, atomic coordinations and bond-angles are intrinsically considered by summing over a large ensemble of atomic configurations from which the ensemble averaged EXAFS spectrum is simulated. This scheme provides a one-to-one matching of experimental data and the corresponding model structure, from which the electronic properties can be calculated in agreement with the measured X-ray-absorption near-edge structure [XANES] spectra. From RMC-EXAFS simulations we show that the disordered structure of *a*WO_3_ comprises mainly corner-sharing and a small proportion of edge-sharing distorted WO_6,5,4_-unit-blocks, while the O atoms hold nearly two-fold coordination with W atoms. The distribution of WO_6,5,4_ polyhedra leads to the formation of spacious channels that could simplify charge-ion injection/diffusion and provide enough free volume for ion storage, which could reduce activation energies and electrode volume variation during ion insertion/extraction. This result supports the fact that the disordered structure of *a*WO_3_ enhances its electrochromic performance when comparing with its crystalline counterparts, as has been assumed in previous studies^10^. Electronic properties in *a*WO_3_ show that the valence band [VB] comprises mainly O-[2*p*] states, while the conduction band [CB] consists mostly of W-[5*d*] states. Disorder-induced localization of electronic states occurs at the O-[2*p*] VB and W-[5*d*] CB tail-states but the density of states [DoS] unveiled a band gap of ≈3.12 eV without defect-induced in-gap states. This suggests that *a*WO_3_ to a great extent retains the electronic structure of its crystalline counterparts. However, from the W-[5*d*-(*t*_2*g*_; *e*_*g*_)] bands derived from the W-*L*_3_ edge XANES spectra a crystal field splitting Δ*d* ≈ 4.0 ± 0.2 eV was found, being it lower than that of crystalline WO_3_.

## Results and Discussion

### Atomic short-range order of *a*WO_3_

**Figure 1:**
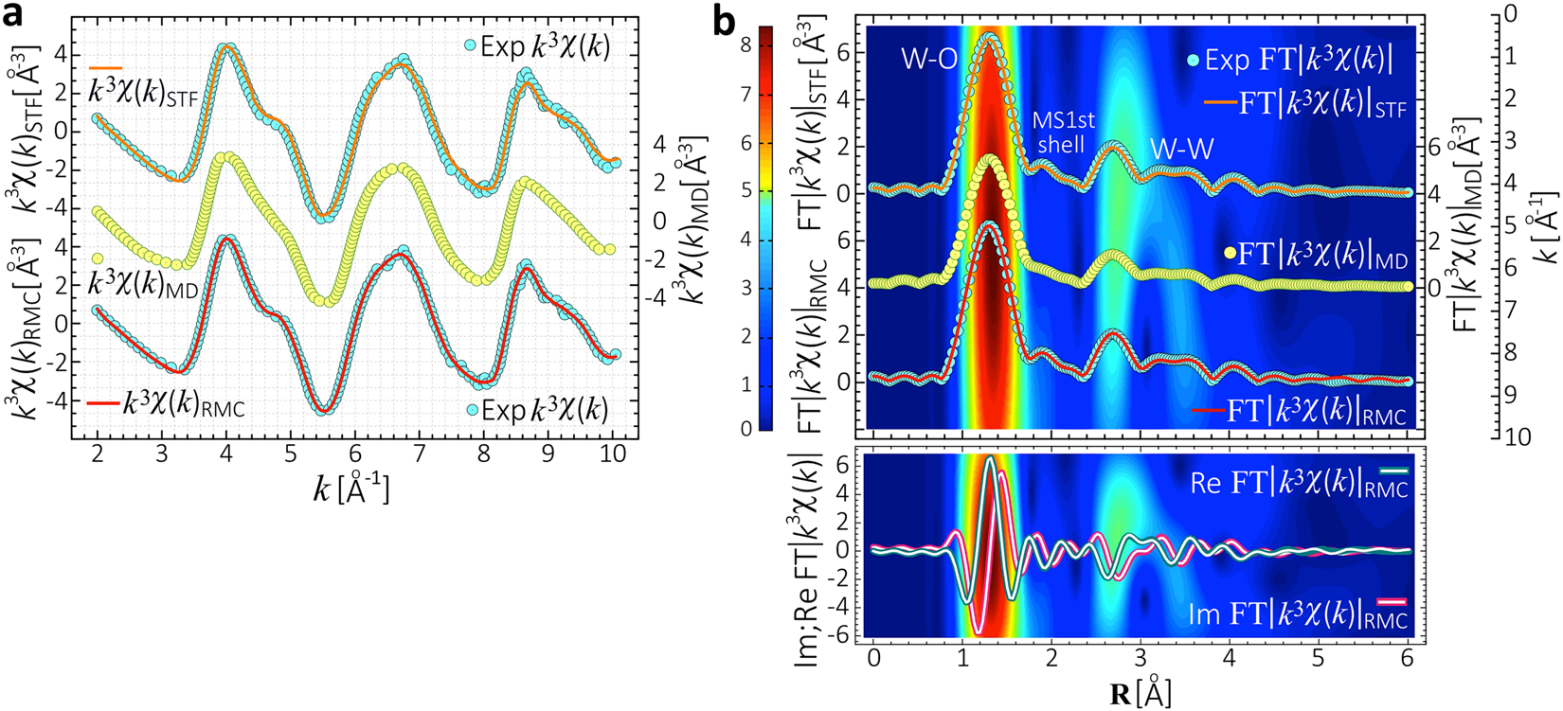
Experimental and simulated EXAFS spectra. (**a**) *Top*: Experimental EXAFS spectrum ${k}^{3}\chi (k)$ of *a*WO_3_ [phase-uncorrected] and its corresponding standard nonlinear least-squares spectral fitting ${k}^{3}\chi {(k)}_{{\rm{STF}}}$. *Middle*: The *ab*-*initio* FMS computed ${k}^{3}\chi {(k)}_{{\rm{MD}}}$ function. *Bottom*: Experimental EXAFS spectrum ${k}^{3}\chi (k)$ of *a*WO_3_ and its corresponding RMC-EXAFS simulated ${k}^{3}\chi {(k)}_{{\rm{RMC}}}$ function. (**b**) *Background*: Wavelet-transform [WT] of the ${k}^{3}\chi (k)$ spectrum. *Top*: The Fourier-transform FT$|{k}^{3}\chi (k)|$ of the experimental EXAFS spectrum and its corresponding standard nonlinear least-squares spectral fitting FT$|{k}^{3}\chi (k){|}_{{\rm{STF}}}$. *Middle*: The *ab*-*initio* FMS computed FT$|{k}^{3}\chi (k){|}_{{\rm{MD}}}$ function. *Bottom*: Experimental FT$|{k}^{3}\chi (k)|$ spectrum and its corresponding RMC-EXAFS simulated FT$|{k}^{3}\chi (k){|}_{{\rm{RMC}}}$ function. *Lower panel*: The Real and Imaginary components for the experimental FT$|{k}^{3}\chi (k)|$ [thick line], and for RMC-EXAFS simulated FT$|{k}^{3}\chi (k){|}_{{\rm{RMC}}}$ spectra [thin line].


As a starting point, standard nonlinear least-squares fitting [STF] of the experimental EXAFS spectrum, ${k}^{3}\chi (k)$, was implemented to obtain preliminary average values for interatomic distances, atomic coordination and *σ* ^2^ parameters [see Methods]. Results from standard fitting ${k}^{3}\chi {(k)}_{{\rm{STF}}}$ displayed in Fig. 1a, show that structural disorder around the photoabsorbing W atoms reduces the oscillation amplitude in the ${k}^{3}\chi (k)$ spectrum at high *k*, thus, main structural features can be identified in the interval Δ*k* ≈ 2–10 Å^−1^. Since the STF approach does not provide any 3D-structure of *a*WO_3_, the experimental ${k}^{3}\chi (k)$ spectrum was compared against the *ab*-*initio* MD-EXAFS, ${k}^{3}\chi {(k)}_{{\rm{MD}}}$ function, calculated directly from MD structural trajectories of *a*WO_3_. To this end, energetically pre-converged *ab*-*initio* MD structural trajectories of *a*WO_3_ comprising W = 64 and O = 192 atoms into cubic cells [V ≈ 4400 Å^3^, *ρ* ≈ 5.27 g/cm^3^] were analyzed, and their associated ${k}^{3}\chi {(k)}_{{\rm{MD}}}$ functions were extracted by *ab*-*initio* self-consistent real-space full multiple-scattering [FMS]^14^ into the muffin-tin approximation [see Methods]. The structural averaging over twelve MD trajectories of *a*WO_3_ leads to a main ${k}^{3}\chi {(k)}_{{\rm{MD}}}$ function properly reproducing the phase, shape, and damping of the oscillations of the experimental ${k}^{3}\chi (k)$ spectrum. However, spectral features at *k* ≈ 4.8 Å^−1^ and *k* ≈ 9.2 Å^−1^ are not well-resolved in the *ab*-*initio* FMS calculated ${k}^{3}\chi {(k)}_{{\rm{MD}}}$ function [Fig. 1a]. Next, in order to correct for those spectral discrepancies and to accurately reproduce the atomic short-range order of *a*WO_3_, those *ab*-*initio* MD structural trajectories were fitted to the experimental ${k}^{3}\chi (k)$ spectrum through RMC-EXAFS simulations [see Methods]^15^. To incorporate the important multiple-scattering processes yielding explicit treatment of three-body correlations, and effects of static disorder due to the fluctuation of interatomic distances, atomic coordination and bond-angles, the following scattering paths between the photoabsorbing  and scattering $\overline{){\rm{\Theta }}};\overline{){\rm{\Phi }}}=$ ; atoms were used; (***i***) single-scattering , (***ii***) triple-scattering , (***iii***) double- and (***iv***) triple-scattering in nearly collinear chains with the photoabsorber at the end of the chain ;  [scattering-angles ≈0°–30°], (***v***) double triangular scattering-path with the photoabsorber at the middle  [scattering-angle≈20°–180°], (***vi***) double-scattering in triangular-paths with scatterers at the 1*st* and 2*nd* shells around the photoabsorber  [scattering-angles ; ≈0°–40°], (***vii***) triple-scattering in collinear chain with scatterers at the 1*st* shell around the photoabsorber  [scattering-angles  ≈10°–180°]. The contribution of each of those scattering-paths to the RMC-EXAFS simulated ${k}^{3}\chi {(k)}_{{\rm{RMC}}}$ spectrum is specified according to ref. 2

1


where *ijn* are serial numbers of  atoms, *ξ* the path-type (***i***)–(***vii***), *θ* is a parametrization angle for (*k*, *θ*), **R**_eff_ is the effective path-length,  the path-legs, (*k*, *θ*) is the EXAFS amplitude and (*k*, *θ*) the phase-shift, *λ* is the mean free path for the photoabsorber  atoms. The amplitude (*k*, *θ*) and phase-shift (*k*, *θ*), for single- and multiple-scattering paths were self-consistently calculated by *ab*-*initio* FMS considering atoms up to **R** ≈ 7 Å from the photoabsorbing  atoms according to


2



3


where (*k*, *θ*), ${{\rm{\Lambda }}}_{{\rm{red}}}(k)$, 2Δ(*k*) and ${\rm{\Phi }}(k,\theta )$ denote the amplitude magnitude, reduction factor, *k*-dependent phase correction and phase^2^. From a first RMC-EXAFS run, the structural averaged ${k}^{3}\chi {(k)}_{{\rm{RMC}}}$ spectrum [Δ*k* ≈ 2–10 Å^−1^], is calculated as a sum of the (*k*) contributions from the different single- and multiple-scattering paths (***i***)–(***vii***), according to


4


Results of spectral fitting from the RMC-EXAFS simulated spectrum, ${k}^{3}\chi {(k)}_{{\rm{RMC}}}$, is in excellent agreement with the experimental ${k}^{3}\chi (k)$ spectrum [Fig. 1a], and as also outlined above, include multiple-scattering events. After convergence, a minimum residual of ≈1–5 × 10^−3^ is attained, which confirms that the RMC-EXAFS refinements correctly reflect the atomic short-range order of *a*WO_3_. Thermal damping of the RMC-EXAFS signal associated to the structural disorder *σ* ^2^, is given by the statistical averaging of ${k}^{3}\chi {(k)}_{{\rm{RMC}}}$ signals obtained by summing over the ensemble of atomic configurations^3^. The Fourier-Transform of the experimental ${k}^{3}\chi (k)$ spectrum into the real-space FT$|{k}^{3}\chi (k)|$ and its corresponding wavelet transform [WT; 2D-contour plot] are shown in Fig. 1b [phase-uncorrected, Real and Im components at the bottom]. The main peak in the FT$|{k}^{3}\chi (k)|$ spectrum at **R** ≈ 1.3 Å with *k* ≈ 5.6 Å^−1^ in the WT, is associated to single-scattering by neighboring O atoms in the first coordination shell [W-O]. The peak in FT$|{k}^{3}\chi (k)|$ at **R** ≈ 2.7 Å with *k* ≈ 4.1 Å^−1^ in the WT, is due to multiple-scattering contributions in the first shell. The peak in FT$|{k}^{3}\chi (k)|$ at **R** ≈ 3.4 Å with *k* ≈ 6.4 Å^−1^ in the WT, emerges from the contribution from mixed single- and multiple-scattering by W and distant O atoms at the second coordination shell. Results from standard nonlinear least-squares fitting FT${|{k}^{3}\chi (k)|}_{{\rm{STF}}}$ of the experimental FT$|{k}^{3}\chi (k)|$ spectrum in Fig. 1b, leads to the structural parameters shown in Table 1. A direct comparison with the *ab*-*initio* FMS calculated FT${|{k}^{3}\chi (k)|}_{{\rm{MD}}}$ function obtained from MD structural trajectories of *a*WO_3_, shows that the FT${|{k}^{3}\chi (k)|}_{{\rm{MD}}}$ signal reproduces the real-space position of the first coordination shell W-O, but the spectral features in the range **R** ≈ 1.7–6.0 Å are not well-resolved [Fig. 1b]. The interatomic bond distances in FT${|{k}^{3}\chi (k)|}_{{\rm{MD}}}$ are close to the experimental data, but the relative intensities of the peaks in FT${|{k}^{3}\chi (k)|}_{{\rm{MD}}}$ are lower than those of the experimental FT$|{k}^{3}\chi (k)|$ spectrum. Thus, main atomic coordination in *ab*-*initio* MD structures of *a*WO_3_ [*N*_W-O_ = 5.38 ± 0.13, *N*_W-W_ = 4.53 ± 0.24], are lower than those obtained from the experimental FT$|{k}^{3}\chi (k)|$ spectrum [*N*_W-O_ = 5.80 ± 0.10, *N*_W-W_ = 5.30 ± 0.10]. Note that STF optimizes the relative weights of scattering-paths, *σ* ^2^ factors and the amplitude reduction factor ${S}_{0}^{2}$ to provide average values of atomic coordination [*N*_W-O_, *N*_W-W_]. To the contrary, atomic coordinations [*N*_W-O_, *N*_W-W_] computed directly from *ab*-*initio* MD structures of *a*WO_3_ correspond to the average over a large ensemble of configurations with different scattering-paths, *σ* ^2^ and ${S}_{0}^{2}$ factors. Since the ${S}_{0}^{2}$ factor entering equation (2) is completely correlated with *N*_W-O_, *N*_W-W_^16^, a small variation in ${S}_{0}^{2}$ could lead to large variation in *N*_W-O_, *N*_W-W_. Thus, differences between the experimental ${k}^{3}\chi (k)$, FT$|{k}^{3}\chi (k)|$ and *ab*-*initio* FMS computed ${k}^{3}\chi {(k)}_{{\rm{MD}}}$, FT${|{k}^{3}\chi (k)|}_{{\rm{MD}}}$ spectra could possibly be ascribed to structural correlations with the ${S}_{0}^{2}$ factor and approximations on the muffin-tin radii for the potential of the photoabsorbing W atoms^14, 16, 17^.

**Table 1:** Main interatomic bond-distances, atomic coordination and 2*σ* ^2^ factors of *a*WO_3_ calculated from nonlinear least-squares fitting of the experimental ${k}^{3}\chi (k)$ and FT$|{k}^{3}\chi (k)|$ spectra to single- and multiple-scattering theory [${S}_{0}^{2}\approx 0.92(4)$, Δ*E*_0_ ≈ 6.84 eV], together with parameters obtained directly from RMC-EXAFS optimized structures of *a*WO_3_.

Least-Squares Fitting		RMC-EXAFS *a*WO_3_	
W-O	1.86 ± 0.02 Å	W-O	1.85 ± 0.03 Å
*N*_W-O_	5.80 ± 0.10	*N*_W-O_	5.74 ± 0.12
${2\sigma }_{{\rm{W}} \mbox{-} {\rm{O}}}^{2}$	0.011(8)	*N*_O-W_	1.91 ± 0.03
W-W	3.74 ± 0.02 Å	W-W	3.73 ± 0.03 Å
*N*_W-W_	5.30 ± 0.10	*N*_W-W_	5.24 ± 0.18 Å
${2\sigma }_{{\rm{W}} \mbox{-} {\rm{W}}}^{2}$	0.136(3)	O-O	2.76 ± 0.03 Å
—	—	*N*_O-O_	7.68 ± 0.21

The RMC-EXAFS refinement Fourier transformed into the real-space FT${|{k}^{3}\chi (k)|}_{{\rm{RMC}}}$ of the W-O and W-W coordination shells in the experimental FT$|{k}^{3}\chi (k)|$ spectrum was calculated according to


5


Spectral fitting from RMC-EXAFS refined FT${|{k}^{3}\chi (k)|}_{{\rm{RMC}}}$ to the experimental FT$|{k}^{3}\chi (k)|$ spectra reproduces the real-space position of the first [W-O] and second coordination shells [W-W], and the multiple-scattering contributions observed in the experimental spectra. These results confirm that the atomic short-range order of *a*WO_3_, can be properly extracted through RMC-EXAFS simulations based on *ab*-*initio* FMS approaches [Fig. 1b, Real and Im components at the bottom]. Table 1 summarizes the main interatomic bond-distances, atomic coordination and *σ* ^2^ factors, obtained from nonlinear least-squares spectral fitting of the measured ${k}^{3}\chi (k)$, FT$|{k}^{3}\chi (k)|$ spectra, and those calculated directly from the RMC-EXAFS optimized structures of *a*WO_3_. The data show an excellent agreement to each other, which confirms that averaging over non-equivalent WO_*x*_ atomic-environments yields accurate reproduction of the ${k}^{3}\chi (k)$, FT$|{k}^{3}\chi (k)|$ spectra and correlated structural parameters of *a*WO_3_. The *σ* ^2^ factors in Table 1 reflect the attenuation of ${k}^{3}\chi (k)$ and FT$|{k}^{3}\chi (k)|$ due to the mean-square static disorder in the distribution of interatomic bond-distances and ionic displacements^3^. Thus, the calculated values for *σ* ^2^ correspond to the degree of disorder in *a*WO_3_, which is mainly manifested in the shortening of interatomic bond-distances [W-O, W-W, O-O], and in the lowering of the atomic coordination [*N*_W-O_, *N*_O-W_, *N*_W-W_, *N*_O-O_], with respect to crystalline phases of WO_3_.

**Figure 2:**
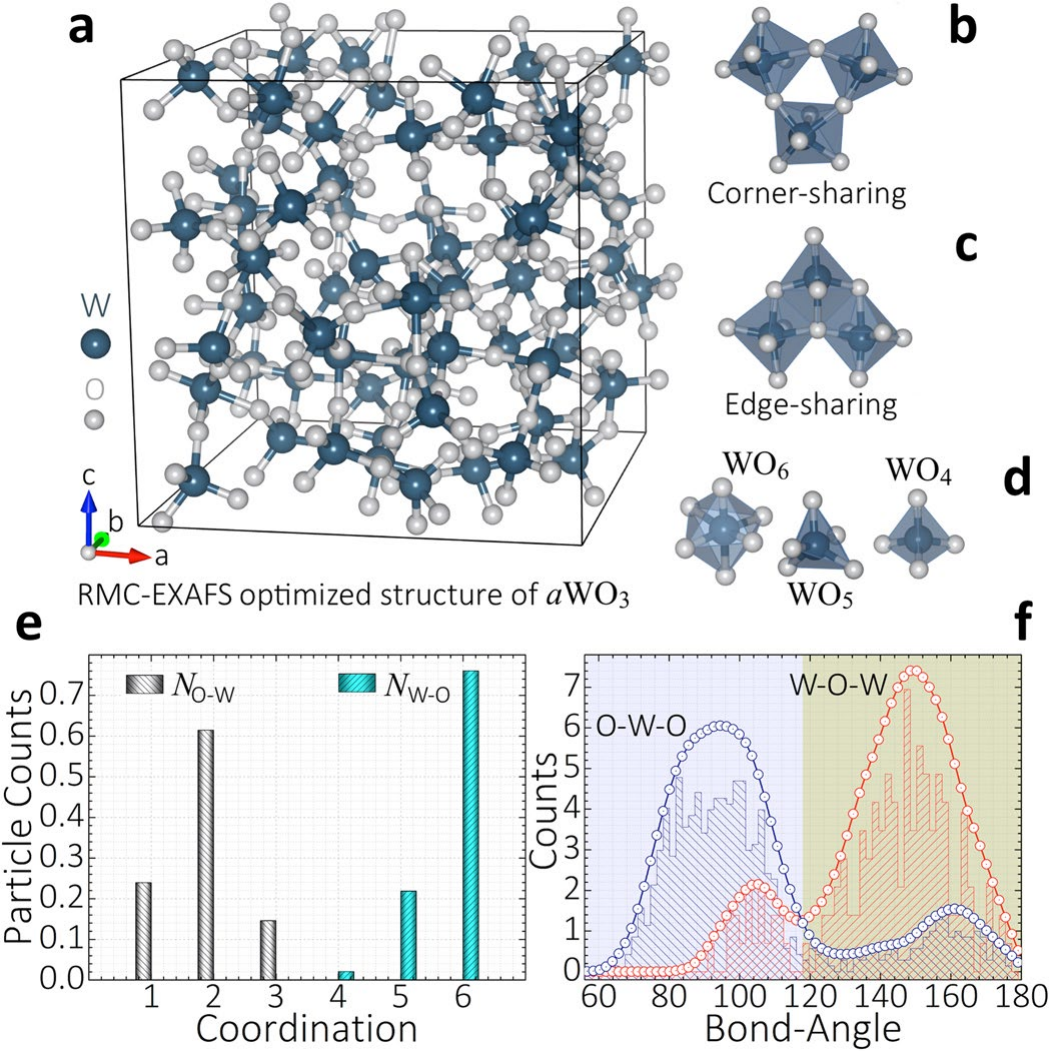
Atomic short-range order of *a*WO_3_. (**a**–**d**) RMC-EXAFS simulated structure of *a*WO_3_ comprising edge- and corner-sharing distorted WO_6,5,4_ units. (**e**) Distribution of atomic coordination yielding a mean value *N*_W-O_ ≈ 5.74 ± 0.12 and *N*_O-W_ ≈ 1.91 ± 0.03. (**f**) Edge-sharing O-W-O and corner-sharing W-O-W bond-angle distributions exhibiting maxima at 94°, 161° and at 105°, 150°, respectively.

To more quantitatively assess the atomic short-range order of our RMC-EXAFS optimized structure of *a*WO_3_ we analyze the local bonding and coordination around the W and O atoms. The RMC-EXAFS optimized structures of *a*WO_3_ comprise mainly corner-sharing distorted WO_6,5,4_ units and a small proportion of edge-sharing WO_6,5,4_ unit-blocks [Fig. 2a–d]. Distribution of atomic coordination [at a W-O bond-length cutoff of ≈2.8 Å], shows that W atoms hold mainly octahedra [*N*_W-O_ = 6, ≈76%], under coordinated pentahedra [*N*_W-O_ = 5, ≈22%], and tetrahedra [*N*_W-O_ = 4, ≈2%] locally bonding with neighbouring O atoms [Fig. 2e]. This results in a main atomic coordination *N*_W-O_ ≈ 5.74 ± 0.12 in the first W-O shell, in agreement with the main value *N*_W-O_ ≈ 5.8 ± 0.1 obtained from least-squares fitting of the ${k}^{3}\chi (k)$ and FT$|{k}^{3}\chi (k)|$ spectra. The O atoms hold nearly two-fold coordination with W atoms, *N*_O-W_ ≈ 1.91 ± 0.10 [Fig. 2e]. The edge-sharing [O-W-O] and corner-sharing [W-O-W] bond-angle distributions show peaks at 94°, 161° and at 105°, 150° [Fig. 2f]. After refinements, all the RMC-EXAFS optimized structures of *a*WO_3_ attained a similar atomic-bonding distribution and displayed small variation in the atomic coordination and bond-angle distributions. This is of course, due to the use of pre-converged MD trajectories, and the structural constraints applied in simulations, which force the input structures to achieve a similar atomic short-range order to properly fit the ${k}^{3}\chi (k)$ and FT$|{k}^{3}\chi (k)|$ spectra [see Methods]. Interatomic bond-lengths were almost the same. Some structures displayed over-coordinated WO_7_-units at larger W-O bond-length cutoff [≈3.12 Å]. However, atomic coordinations *N*_W-O_ = 5,6 dominates accurately the structural characteristics of the EXAFS spectra of *a*WO_3_. Here the reported structural correlations and physical quantities correspond to the average over twelve RMC-EXAFS optimized structures of *a*WO_3_.

**Figure 3:**
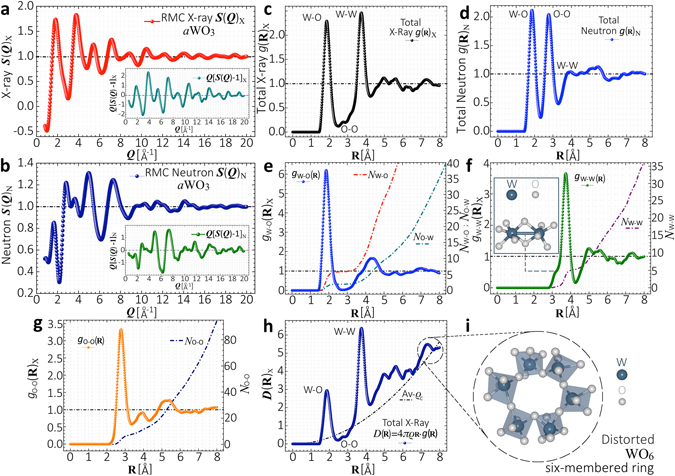
Structure pair correlation functions. (**a**) Calculated X-ray ${\boldsymbol{S}}{({\boldsymbol{Q}})}_{{\rm{X}}}$ and (**b**) Neutron ${\boldsymbol{S}}{({\boldsymbol{Q}})}_{{\rm{N}}}$ structure factors from RMC-EXAFS simulated structures of *a*WO_3_. Insets display the reduced structure function ***Q***[${\boldsymbol{S}}({\boldsymbol{Q}})$−1]. (**c**) Calculated total X-ray $g{({\bf{R}})}_{{\rm{X}}}$, and (**d**) Neutron $g{({\bf{R}})}_{{\rm{N}}}$ pair distribution functions. (**e**–**g**) X-ray partial distribution functions *g*_W-O_(**R**), *g*_W-W_(**R**), *g*_O-O_(**R**) and corresponding atomic coordination *N*_W-O_, *N*_O-W_, *N*_W-W_, *N*_O-O_. Inset in (**f**) shows a W-W pair with bond-length **R** ≈ 3.1 Å, in neighboring edge-sharing WO_*x*_ structural units. (**h**) Total X-ray ***D***(**R**) = 4*πρ* **R** · *g*(**R**), the dotted line corresponds to the average density contribution [Av-*ρ*_*c*_]. (**i**) Formation of WO_6_ six-membered rings associated to the peak at **R** ≈ 7.3 Å in the ***D***(**R**) of (**h**), and in $g{({\bf{R}})}_{{\rm{X}}}$, $g{({\bf{R}})}_{{\rm{N}}}$ of (**c**–**g**).

To compare our results with X-ray and neutron diffraction experiments, the atomic short-range order of our RMC-EXAFS optimized structures of *a*WO_3_ was further analyzed in terms of the structure factor ***S***(***Q***), defined according to

6 $${\boldsymbol{S}}({\boldsymbol{Q}})=\frac{1}{N}\sum _{j,k}{b}_{j}{b}_{k}\langle {e}^{i{\boldsymbol{Q}}[{{\bf{R}}}_{j}-{{\bf{R}}}_{k}]}\rangle =1+4\pi \rho {\int }_{0}^{\infty }{{\bf{R}}}^{2}\frac{\sin ({\boldsymbol{Q}}{\bf{R}})}{{\boldsymbol{Q}}{\bf{R}}}[g({\bf{R}})-1]d{\bf{R}},$$

where *b*_*j*_ is the X-ray/neutron scattering length, **R**_*j*_ the position of the atom *j*, and *N* the number of atoms. The X-ray ${\boldsymbol{S}}{({\boldsymbol{Q}})}_{{\rm{X}}}$, neutron ${\boldsymbol{S}}{({\boldsymbol{Q}})}_{{\rm{N}}}$ structure factors and their associated reduced structure function ***Q***[***S***(***Q***)−1], exhibit interference maxima around scattering wavevector magnitudes in the interval Δ***Q*** ≈ 1.5–8.2 Å^−1^. Beyond that range the oscillation amplitude in ${\boldsymbol{S}}{({\boldsymbol{Q}})}_{{\rm{X}}}$ and ${\boldsymbol{S}}{({\boldsymbol{Q}})}_{{\rm{N}}}$ is damped out due to the structural disorder [Fig. 3a,b]. ${\boldsymbol{S}}{({\boldsymbol{Q}})}_{{\rm{X}}}$ and ${\boldsymbol{S}}{({\boldsymbol{Q}})}_{{\rm{N}}}$ exhibit distinct interference patterns caused by different scattering processes. The scattering due to W atoms contributes more to ${\boldsymbol{S}}{({\boldsymbol{Q}})}_{{\rm{X}}}$ than that from O atoms, because X-rays interact mainly with the electron cloud surrounding the atoms [*Z*-dependent]. Contrary, the scattering due to O atoms contributes more to ${\boldsymbol{S}}{({\boldsymbol{Q}})}_{{\rm{N}}}$ than that of W atoms, because neutrons interact with the atomic nucleus. These distinct features, the relative amplitude, peak position and the line-shape in our calculated ${\boldsymbol{S}}{({\boldsymbol{Q}})}_{{\rm{X}}}$, ${\boldsymbol{S}}{({\boldsymbol{Q}})}_{{\rm{N}}}$ and ***Q***[***S***(***Q***)−1] patterns agree with previous data reported for stoichiometric W-based oxides from X-ray, electrons and neutron diffraction experiments^18–22^, and show a qualitative similarity to data for sub-stoichiometric W oxide^23^.

Those structural characteristics are more clearly observed in the total X-ray $g{({\bf{R}})}_{{\rm{X}}}$ and neutron $g{({\bf{R}})}_{{\rm{N}}}$ PDF defined according to

7 $$g({\bf{R}})=\frac{{\sum }_{\alpha ,\beta }{c}_{\alpha }{b}_{\alpha }{c}_{\beta }{b}_{\beta }{g}_{\alpha \beta }({\bf{R}})}{{({\sum }_{\alpha }{c}_{\alpha }{b}_{\alpha })}^{2}},$$

where *c*_*α*_, *c*_*β*_ are the concentrations of *α*, *β* atoms [*c*_*α*,*β*_ = *N*_*α*,*β*_/*N*], *b*_*α*_, *b*_*β*_ denotes the X-ray/neutron scattering length of species *α*, *β*, and ${g}_{\alpha \beta }({\bf{R}})$ denotes the partial PDF according to

8 $${g}_{\alpha \beta }({\bf{R}})=\frac{1}{4\pi {{\bf{R}}}^{2}\rho {c}_{\beta }}\frac{d\langle {n}_{\alpha \beta }({\bf{R}})\rangle }{d{\bf{R}}}=\frac{1}{4\pi {{\bf{R}}}^{2}{N}_{\beta }}\frac{d\langle {n}_{\alpha \beta }({\bf{R}})\rangle }{d{\bf{R}}},$$

with $d\langle {n}_{\alpha \beta }({\bf{R}})\rangle$ the ensemble average number of *β* atoms in a shell *d* **R** at a distance **R** of an *α* atom. Here *ρ* is the number density and ${g}_{\alpha \beta }({\bf{R}})$ is the probability to find a *β* atom at a distance **R** from an *α* atom. The calculated $g{({\bf{R}})}_{{\rm{X}}}$ exhibits two peaks at **R** ≈ 1.85 Å, **R** ≈ 3.7 Å, associated to the first [W-O], [W-W] coordination shells, respectively. The first [O-O] shell at **R** ≈ 2.75 Å is also resolved in $g{({\bf{R}})}_{{\rm{X}}}$, but it exhibits lower intensity [Fig. 3c]. Contrary, $g{({\bf{R}})}_{{\rm{N}}}$ shows two peaks at **R** ≈ 1.85 Å, **R** ≈ 2.75 Å, associated to the first [W-O], [O-O] coordination shells, but the [W-W] shell at **R** ≈ 3.7 Å, is not well-resolved in $g{({\bf{R}})}_{{\rm{N}}}$ [Fig. 3d]. In Fig. 3e–g, maxima in the partials *g*_W-O_ ${({\bf{R}})}_{{\rm{X}}}$ [at **R** ≈ 1.85, **R** ≈ 3.58, **R** ≈ 4.36 Å], in *g*_O-O_(**R**)_X_ [at **R** ≈ 2.75, **R** ≈ 3.90, **R** ≈ 5.26 Å], and in *g*_W-W_(**R**)_X_ [at **R** ≈ 3.7, **R** ≈ 4.96, **R** ≈ 5.6 Å], relate to the 1*st*, 2*nd* and 3*rd* coordination shells [W-O, O-O, W-W], respectively. Note that the main atomic coordinations [*N*_W-O_, *N*_O-W_, *N*_W-W_, *N*_O-O_] as a function of **R**, and calculated according to

9 $${N}_{\alpha \beta }({\bf{R}})=4\pi \rho {c}_{\beta }{\int }_{0}^{{\bf{R}}}{g}_{\alpha \beta }({\bf{R}})\,{{\bf{R}}}^{2}d{\bf{R}},$$

at the first minimum of *g*_W-O_(**R**)_X_, *g*_W-W_(**R**)_X_, *g*_O-O_(**R**)_X_, in Fig. 3e–g agree with the main values *N*_W-O_ ≈ 5.8 ± 0.1 and *N*_W-W_ ≈ 5.3 ± 0.1, obtained by least-squares fitting of the ${k}^{3}\chi (k)$ and FT$|{k}^{3}\chi (k)|$ spectra [Table 1]. The low intensity shoulder at the left side of *g*_W-W_(**R**)_X_ is due to W-W pairs with bond-length **R** ≈ 3.1 Å, in neighboring edge-sharing WO_*x*_ structural units [inset Fig. 3f]. Those short W-W bonds induce small polaron formation upon insertion of oxygen-vacancies [*O*_*v*_], or alkali metal impurity ions [Li^+^] and charge-balancing electrons^10, 24^. It has been suggested that electrochromism in *a*WO_3_ arises from the optical absorption due to small polaron hopping associated to the formation of W^5+^ states due to transfer of electrons from *O*_*v*_-sites and inserted Li^+^ species^10, 24^. Short W-W bonds have been previously reported for ion exchange and sputtered *a*WO_3_ solid thin film oxides^18, 20^.

The RMC-EXAFS simulations on *a*WO_3_ not only fit the experimental ${k}^{3}\chi (k)$ and FT$|{k}^{3}\chi (k)|$ spectra, but also the calculated X-ray ***D***(**R**) = 4*πρ* **R** · *g*(**R**) function [Fig. 3h], accurately reproduces the relative amplitude, peak position and the line-shape of earlier ***D***(**R**)’s reported from electron and X-ray diffraction experiments^13, 18–22^. Especially, the peak at **R** ≈ 7.3 Å in the ***D***(**R**) of the RMC-EXAFS optimized structures of *a*WO_3_ accurately reproduces the formation of WO_6_ six-membered rings [Fig. 3i], in agreement with experimental data^18, 21, 22^. Particularly, the distribution of distorted WO_*x*_ octahedra-units in the structure of *a*WO_3_ leads to the formation of distorted WO_*x*_-like chains analogous to those found in the Magnéli phases W_*x*_O_*z*_^25^, and a wide distribution of WO_4,5,6,7_-membered rings comprising distinct WO_*x*_ units with large free volume forming spacious channels as shown in Fig. 3i. Analogous channels have been reported for hexagonal *h*-WO_3_ and nanostructured WO_3_ systems, which have WO_6_-three- and six-membered rings forming trigonal cavities with hexagonal- and four-coordinated square channels^26, 27^. Those local structures provides large available sites for cation intercalation and superior charge densities, simplifying charge-ion injection/diffusion and providing enough free volume for ion storage, which reduce activation energies and electrode volume variation during ion insertion/extraction. These results support the fact that the disordered structure of *a*WO_3_ enhances its electrochromic performance when comparing with its crystalline counterparts, as has been assumed in previous technical studies^10^. Formation of different distorted WO_*x*_-like chains and WO_4,5,6,7_-membered rings comprising distinct WO_*x*_ units suggest that the atomic short-range order of *a*WO_3_ should consist of a mixture of the different symmetries existing in the polymorphs and Magnéli phases of WO_3_, rather than comprising a single hexagonal or distorted ReO_3_-octahedra phase, as suggested previously^13, 18, 22^. This would also explain why *a*WO_3_ turns into a mixture of the monoclinic, hexagonal and triclinic phases of crystalline WO_3_ upon heating^28^. Difficulties with the assignment of a single phase to *a*WO_3_, arises because previous structural models were deduced by hand from direct comparison of the ***D***(**R**)’s of crystalline and *a*WO_3_. Thus, such model results are unrealistic since they do not take into account the contribution of distinct atomic environments to the total ***D***(**R**) of *a*WO_3_. Structural characterizations of *a*WO_3_ in terms of crystalline phases are ambiguous because the monoclinic/hexagonal WO_3_, and the W_*x*_O_*z*_-like Magnéli phases exhibit very analogous ***D***(**R**) functions^23^. Because of the local-structural reconstruction at nonequivalent atomic environments, amorphous materials should display a distribution of interatomic distances, and lower average atomic coordination. Here, energetically and structurally pre-converged MD structural trajectories of *a*WO_3_ were used. This ensures that effects of static disorder due to the fluctuation of interatomic distances, atomic coordination and bond-angles, were intrinsically taken into account in the simulations. Multiple-scattering processes were included by self-consistent calculations considering atoms beyond the first coordination shell. Thus, pre-converged amplitudes and phase-shifts for different scattering processes around the photoabsorbing W atoms, allow the explicit treatment of three-body correlations. The use of bond-distance and coordination constraints prevent the *a*WO_3_ simulated structures getting away from the atomic short-range order defined in the measured ${k}^{3}\chi (k)$ and FT$|{k}^{3}\chi (k)|$ spectra. Thus, since the structure was optimized at each atomic displacement, until it reached an accurate one-to-one matching with the experimental ${k}^{3}\chi (k)$ and FT$|{k}^{3}\chi (k)|$ spectra, we expect our RMC-EXAFS-based simulation to properly describe the atomic short-range order of *a*WO_3_.

### Electronic properties of *a*WO_3_

**Figure 4:**
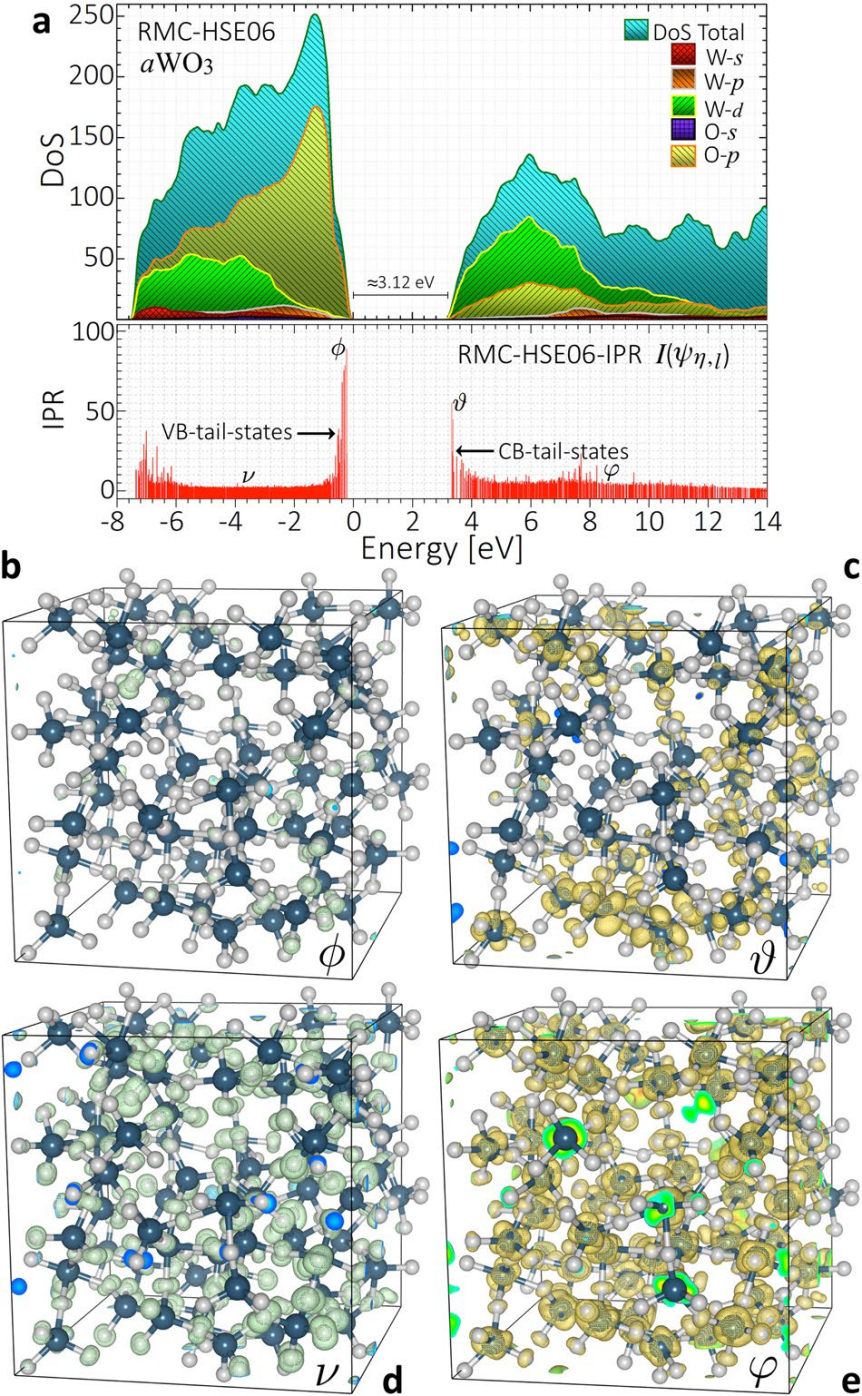
Electronic properties of *a*WO_3_. (**a**) Total and projected DoS for RMC-EXAFS optimized structures of *a*WO_3_ and corresponding IPR, $I({\psi }_{\eta ,l})$ function. (**b**,**c**) Electronic charge density contributions [Isosurfaces] to the localized VB and CB tail-states [$\varphi$, $\vartheta$], and (**d**,**e**) delocalized Bloch-like VB and CB states [$\nu$, $\phi$], defined in Fig. 4(a).

We now use the RMC-EXAFS optimized structures of *a*WO_3_ to assess the correlations between atomic short-range order and the electronic properties by detailed calculations of the electronic structure by hybrid density functional theory [DFT]^29^, and electronic transitions associated to the XANES spectra by *ab*-*initio* finite difference methods [FDM]^30^ [see Methods]. Figure 4a displays the total and projected DoS of *a*WO_3_. The VB comprises mostly O-[2*p*] states while the CB consists mainly of W-[5*d*] states. This suggests that *a*WO_3_ to a great extent conserves the electronic structure defined in its crystalline counterparts. From the RMC-EXAFS optimized structures of *a*WO_3_ a band gap of ≈3.12 eV without defect-induced in-gap states in the DoS was consistently calculated. Increasing the HF exchange in the hybrid HSE06 functional yielded a band gap lowering of ≈0.8 eV. Note that the lack of periodicity in *a*WO_3_ yields an ill-defined k-vector, thus, the electronic energy band gap is merely given by the Kohn-Sham eigenvalue difference between highest occupied [HOMO] and lowest unoccupied [LUMO] states. When going from crystalline WO_3_ [band gap ≈ 2.8 eV^31^], to *a*WO_3_ [band gap ≈ 3.12 eV], a band gap widening of ≈0.32 eV is found. This electronic energy band gap is in good agreement with our experimental optical band gap of $\hslash {\omega }_{g}$ ≈ 3.2 ± 0.07 eV, previously reported for sputtered *a*WO_3_ thin-film oxides from optical UV-vis-NIR spectroscopy^24^. To describe the disorder-induced localization of electronic states at the VB and CB tails-states the inverse participation ratio [IPR; $I({\psi }_{\eta ,l})$], which allows to distinguish between localized and delocalized states, was calculated according to

10 $$I({\psi }_{\eta ,l})=N\frac{{\sum }_{i=1}^{N}{|{\psi }_{\eta ,l}({{\bf{R}}}_{i})|}^{4}}{{[{\sum }_{i=1}^{N}{|{\psi }_{\eta ,l}({{\bf{R}}}_{i})|}^{2}]}^{2}}$$

where ${\psi }_{\eta ,l}({{\bf{R}}}_{i})$ denotes the eigenstate projection of a state $\eta$ for the atom at a distance **R**_*i*_, and angular momentum *l*. *N* is the total number of atoms in the cell. For an ideally localized state, only one atomic site contributes all the charge [$I({\psi }_{\eta ,l})$ = 1]. For a uniformly delocalized state, the charge contribution per site is uniform and equal to 1/*N* [$I({\psi }_{\eta ,l})\approx \mathrm{1/}N$]. Thus, large $I({\psi }_{\eta ,l})$ values correspond to localized states, and low $I({\psi }_{\eta ,l})$ to delocalized states^32^.

The $I({\psi }_{\eta ,l})$ function based on the electron density obtained from the hybrid DFT calculation is shown at the bottom of Fig. 4a. For RMC-EXAFS refined *a*WO_3_, $I({\psi }_{\eta ,l})$ exhibits high values at the VB and CB edges, suggesting electronic localization of the O-[2*p*] VB and W-[5*d*] CB tail states. The VB tail-states shows slightly more localization than those electronic orbitals at the CB tail states. The mobility band gap defined as the energy gap between extended VB and CB states, is estimated to be ≈3.22 eV. The extent of localization of the VB and CB tail states in *a*WO_3_ depends on the charge density contribution due to the atomic short-range order of distinct WO_*x*_-units. Figures 4b,c display the electronic charge density contribution arising from localized O-[2*p*] VB and W-[5*d*] CB tail states [denoted $\varphi$; $\vartheta$ in Fig. 4a]. Fully delocalized Bloch-like VB and CB states [denoted $\nu$; $\phi$], are also shown in Fig. 4d,e. Localized VB tail states arise from O-[2*p*]-like charge density contributions due to single- and two-coordinated O atoms holding short bonds with the W atoms [≈1.72–1.76 Å]. Localized CB tail states arise from W-[5*d*]-like charge density contributions due to undercoordinated WO_4,5_-units, and to a minor extent to formally sixfold-coordinated W atoms that are largely displaced from the center of the WO_6_-octahedra. Unpaired electrons from the single-coordinated O ions could yield localized acceptors-like dangling bonds at the VB tail edge. Overcoordinated W atoms could yield localized donor-like electronic states at the CB tail-edge. Therefore, the VB and CB edges are strongly dependent on the type of ligands and on the atomic short-range order around the O and W sites in the first and second coordination shells.

**Figure 5:**
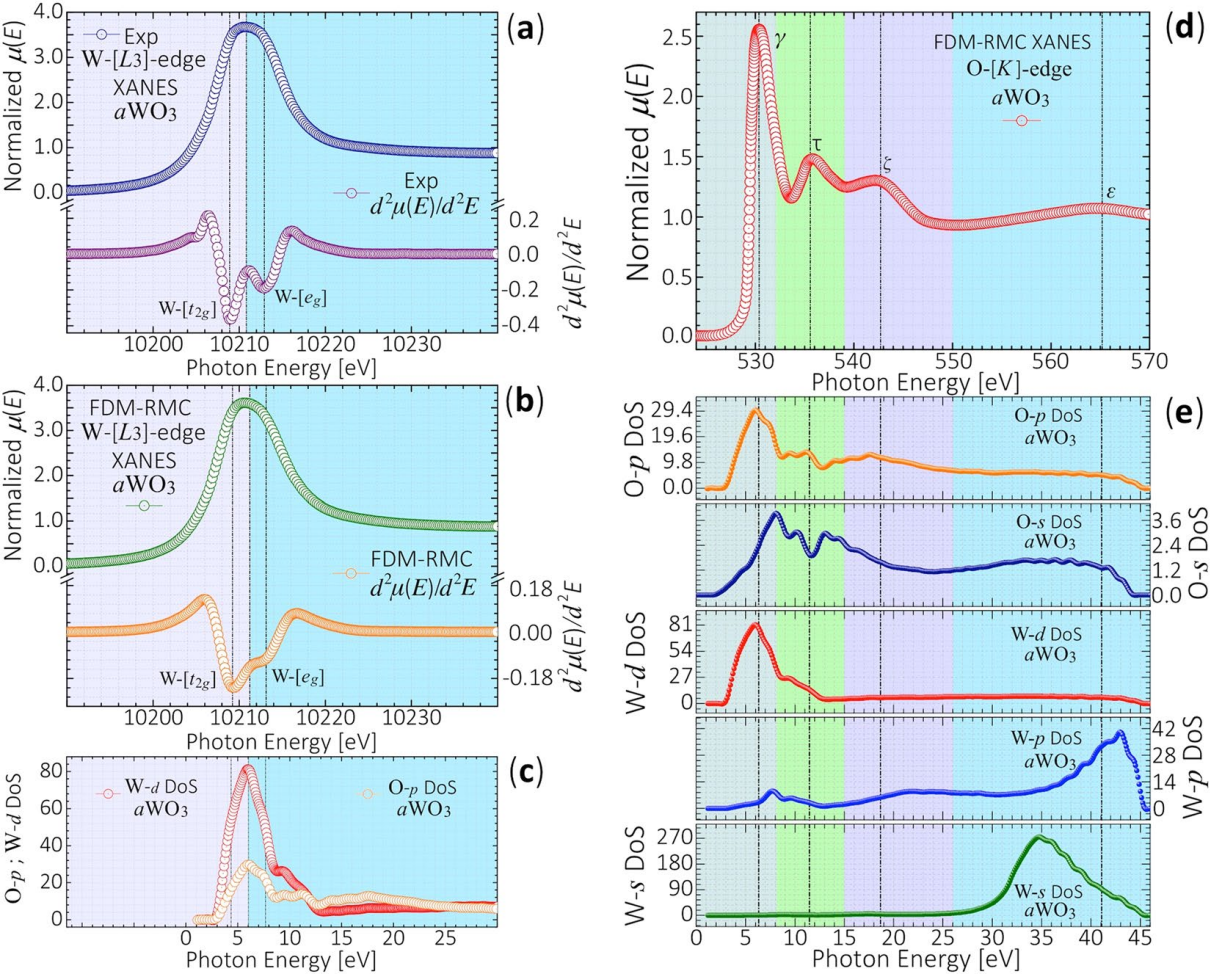
Normalized W-*L*_3_-edge and O-*K*-edge XANES spectra of *a*WO_3_. (**a**) Normalized experimental W-*L*_3_-edge XANES spectrum of *a*WO_3_, and its second derivative ${d}^{2}\mu (E)/{d}^{2}E$. (**b**) *Ab*-*initio* FDM computed W-*L*_3_-edge XANES spectrum from RMC-EXAFS optimized structures of *a*WO_3_, and its second derivative ${d}^{2}\mu (E)/{d}^{2}E$. The spectrum exhibits absorption maxima associated to electronic transitions of the photoelectron from the initial W-[2*p*_3/2_] orbitals to the final unoccupied W-[5*d*]-O-[2*p*] hybridized CB states. From the relative energy separation of the W-[*t*_2*g*_] and W-[*e*_*g*_] bands in the second derivative ${d}^{2}\mu (E)/{d}^{2}E$ of the W-*L*_3_-edge XANES spectra, a crystal field splitting Δ*d* ≈ *E*(*e*_*g*_) − *E*(*t*_2*g*_) ≈ 4.0 ± 0.2 eV is found. (**c**) Contribution of the W-[5*d*] and O-[2*p*] projected DoS to the W-*L*_3_-edge XANES spectrum of *a*WO_3_. (**d**) *Ab*-*initio* FDM calculated O-*K*-edge XANES spectrum of *a*WO_3_, as computed from its RMC-EXAFS optimized structures. The spectrum is due to electronic transitions from the O-[1*s*] core-level into unoccupied O-[2*p*] orbitals. (**e**) Contribution of the W-[*s*, *p*, *d*] and O-[*s*, *p*] projected DoS to the calculated O-*K*-edge XANES spectrum of *a*WO_3_.

Figure 5a,b display the experimental and *ab*-*initio* FDM-RMC computed W-*L*_3_-edge XANES spectra of *a*WO_3_. The normalized spectra exhibit a strong and broad white-line absorption maximum above the absorption edge-energy centered at ≈10210.8 eV. The relative intensity, energy position, line shape and the electronic transitions in the experimental spectrum are well reproduced by the FDM-XANES function calculated from the RMC-EXAFS optimized structures of *a*WO_3_. According to the dipole selection rules, the absorption W-*L*_3_-edge is due to allowed electronic dipole transitions of the photoelectron from the initial W-[2*p*_3/2_] orbitals to the final unoccupied hybridized W-[5*d*]-O-[2*p*] CB states. Thus, from the projected DoS it is qualitatively observed that the W-*L*_3_-edge XANES spectrum of *a*WO_3_ follows the distribution of W-[5*d*] and O-[2*p*] CB orbitals [Fig. 5c]. The second derivative, ${d}^{2}\mu (E)/{d}^{2}E$, of the measured W-*L*_3_-edge XANES spectrum [bottom Figs. 5a,b] exhibits lower and higher energy minima at ≈10208.9 eV and at ≈10212.9 eV, due to the splitting of the W-[5*d*] orbitals into the W-[*t*_2*g*_] and W-[*e*_*g*_] bands by the crystal field of the surrounding O atoms. In the calculated ${d}^{2}\mu (E)/{d}^{2}E$ of the W-*L*_3_-edge FDM-XANES spectra the minima associated to the W-[*t*_2*g*_] and W-[*e*_*g*_] bands are located at ≈10209.2 eV and ≈10213.2 eV, respectively. The W-[*e*_*g*_] orbitals tend to be smeared out, broadened and shifted by ≈0.3 eV, relative to the experimental spectrum. From the relative energy separation of the W-[*t*_2*g*_] and W-[*e*_*g*_] bands, the crystal field splitting is found to be Δ*d* ≈ *E*(*e*_*g*_) − *E*(*t*_2*g*_) ≈ 4.0 ± 0.2 eV, being lower than that of crystalline WO_3_^33^. Considering that the W-[*e*_*g*_] orbitals point toward neighboring O-[2*p*] orbitals, then this weaker crystal-field splitting Δ*d* could be ascribed to the local structural disorder in the first and second coordination shells along with the contribution of undercoordinated W and O atoms [*N*_W-O_ ≈ 5.74 ± 0.12, *N*_O-W_ ≈ 1.91 ± 0.03].

Since the electronic correlations due to the interaction between the core-hole and the excited electron are small at the O-*K*-edge, the projected DoS, which reflects the electronic ground state, provides a consistent interpretation of the O-*K*-edge XANES spectra of *a*WO_3_. Figure 5d shows the *ab*-*initio* FDM calculated O-*K*-edge XANES spectrum of *a*WO_3_ as calculated from the RMC-EXAFS optimized structures. The calculated O-*K*-edge FDM-XANES spectrum properly reproduces the relative intensity, energy position and shape of earlier reported spectra for *a*WO_3_^34^. In the framework of the dipole selection rules, the spectrum is due to electronic transitions from the O-[1*s*] core-level into the unoccupied O-[2*p*] orbitals. Due to the electronic hybridization between the W-[5*d*] and O-[2*p*] orbitals, the O-*K*-edge also provides the features of the density of W-[5*d*] states. The O-*K*-edge XANES spectrum in Fig. 5d shows a main peak at *γ* ≈ 530.2 eV. From the projected DoS in Fig. 5e, it is noted that this peak reflects the O-[2*p*] states in the *t*_2*g*_ CB due to unoccupied W-[5*d*] and O-[2*p*] orbitals. It has been argued that the relative intensity and width of the *γ* peak is determined by the number of O-[2*p*] empty states, and by the width of the W-[5*d*]-[*t*_2*g*_] band, respectively. This depends on the contribution of nonequivalent O atoms in the first coordination shell [W-O]^34^. The peak at *τ* ≈ 535.7 eV emerges from W-[5*d*] [*e*_*g*_]-O[2*p*] hybridization while the peak *ζ* ≈ 542.8 eV is due to W-[6*sp*]-O-[2*p*] interactions. The relative intensity of the *τ*-*ζ*-peaks depends on the atomic short-range order of the WO_*x*_-units present in the structure of *a*WO_3_, and thus, the *ζ*-peak exhibits lower intensity when comparing with crystalline WO_3_^34^. The CB states of the O projected DoS in Fig. 5e, suggests that the O-*K*-edge XANES spectrum of *a*WO_3_, which gives the unoccupied final states located above the Fermi level, emerges mainly from contributions due to the W-[5*d*]-O-[2*p*] hybridized states. The W-[*s*, *p*] states do not contribute significantly to the absorption at low energies, but they contribute to the *ζ*-peak, and also contribute in some extent to the broad feature at *ε* ≈ 565.1 eV, observed in the O-*K*-edge XANES spectrum of *a*WO_3_.

Finally, we remark that our scheme could offer a consistent route to experimentally and theoretically unveil the atomic short-range order of aTMOs, and how local disorder affects their underlying electronic properties. The approach provides a one-to-one matching of experimental data and corresponding model structure from which electronic properties can be directly calculated in agreement with the electronic transitions giving rise to the XANES spectrum of aTMOs.

## Methods

### Thin-film oxide deposition and X-ray-absorption spectroscopy [XAS] experiments

In this study we used *a*WO_3_ thin film oxides [thickness 600 ± 20 nm, density *ρ* ≈ 5.27 g/cm^3^] deposited by reactive DC magnetron sputtering, with an O/W ratio of 3.00 ± 0.04 as determined by Rutherford Backscattering Spectrometry [RBS], and previously reported^24^. X-ray-absorption near-edge structure [XANES], and extended X-ray-absorption fine structure [EXAFS] spectra at the W-*L*_3_-edge of *a*WO_3_ thin-films oxides were collected using a passivated implanted planar silicon [PIPS] detector in fluorescence mode at beamline I811-MAX-lab synchrotron source, Lund, Sweden^35^. The beam was focused using a Si[111] double-crystal monochromator. A total of ten EXAFS spectra, ${k}^{3}\chi (k)$, were extracted from standard data reduction, absorption edge energy calibration, and background subtraction, as implemented in ATHENA^36^. Those spectra were averaged to a total ${k}^{3}\chi (k)$ spectrum in the range Δ*k* ≈ 2–10 Å^−1^, and then Fourier transformed into the real-space FT$|{k}^{3}\chi (k)|$ in the interval Δ**R** ≈ 0–6 Å. Standard nonlinear least-squares EXAFS-fitting was implemented to previously obtain main values for interatomic distances, coordination numbers and *σ* ^2^ factors. To this end, atomic clusters of *a*WO_3_ generated by ATOMS^36^, were fitted to the experimental ${k}^{3}\chi (k)$ [Δ*k* ≈ 2–10 Å^−1^], and FT$|{k}^{3}\chi (k)|$ [Δ**R** ≈ 0–6 Å] spectra by ARTEMIS^36^. Amplitude and phase shift for single [W-O, W-W] and multiple scattering [W-O-O, W-W-O, W-O-W-O] paths, were calculated self-consistently by the *ab*-*initio* FMS FEFF8.4 code^14^. Fitting was carried out by allowing small variations in the interatomic distances and atomic coordination, while the *σ* ^2^ factors and the threshold energy shift Δ*E*_0_ were treated as free parameters.

### *Ab*-*initio* molecular dynamics MD simulations and FMS calculations of EXAFS spectra

In order to generate energetically and structurally pre-converged 3D-models of *a*WO_3_ for further RMC-EXAFS refinements, *ab*-*initio* MD simulations, as implemented in VASP^29^, were carried out to extract snapshots of structural trajectories of *a*WO_3_. To this end, a cubic cell comprising W = 64 and O = 192 atoms [V ≈ 4400 Å^3^, *ρ* ≈ 5.27 g/cm^3^], rescaled from the crystalline symmetry of monoclinic WO_3_ [space group P121/*n*1; ICSD 14332], was used as input structure. Amorphization was carried out by melting the cubic cell by heating it up to 5000 K [WO_3_ melting point 1743 K]. The MD was equilibrated in the liquid state for 2 ps, and then, allowed to evolve for 2 ps, using 1 fs time steps at constant energy as a micro-canonical ensemble. Twelve MD snapshots were selected and quenched down to 300 K, to simulate *a*WO_3_. Reaching a steady-state condition upon 10^4^ ionic steps ensures that the MD was energetically and structurally relaxed. The Perdew-Burke-Ernzerhof [PBE]^37^ exchange-correlation potential was used with a plane wave cutoff energy of 700 eV, and atomic positions were optimized by a force convergence criterion of 0.01 eV/Å. We used these energetically and structurally pre-converged MD structural trajectories of *a*WO_3_ because they resemble more closely the experimental EXAFS spectrum, being computationally more efficient for RMC-EXAFS optimization. To assess the reliability of the MD structural trajectories of *a*WO_3_, MD-EXAFS functions ${k}^{3}\chi {(k)}_{{\rm{MD}}}$ were computed by *ab*-*initio* FMS and compared against the measured ${k}^{3}\chi (k)$ spectra. The complex exchange-correlation Hedin-Lundqvist self-energy potential was used. The scattering potentials were computed in the muffin-tin approximation and muffin-tins were overlapped to 1.15 to reduce effects due to potential discontinuities using FEFF8.4^14^. An amplitude reduction factor ${S}_{0}^{2}\approx 0.90(2)$ was estimated from the overlap integral by self-consistent calculations of the cluster potential. The thermal damping of the MD-EXAFS signals due to the structural disorder *σ* ^2^, is given by the statistical averaging of ${k}^{3}\chi {(k)}_{{\rm{MD}}}$ signals obtained by summing over the ensemble of twelve MD trajectories. Single W-*L*_3_-edge ${k}^{3}\chi {(k)}_{{\rm{MD}}}$ functions were computed for each photoabsorbing W atom inside clusters of radii **R** = 7 Å, and then averaged to a total ${k}^{3}\chi {(k)}_{{\rm{MD}}}$ and Fourier transformed to a total FT${|{k}^{3}\chi (k)|}_{{\rm{MD}}}$ function.

### Reverse Monte Carlo simulations RMC-EXAFS

To simulate the atomic short-range order of *a*WO_3_, the twelve MD structural trajectories of *a*WO_3_ were fitted to the experimental ${k}^{3}\chi (k)$ [Δ*k* ≈ 2–10 Å^−1^] and FT$|{k}^{3}\chi (k)|$ [Δ**R** ≈ 0–6 Å] spectra by RMC-EXAFS simulations, as implemented in RMCProfile^15^. From the prior EXAFS analysis by ARTEMIS, the threshold energy shift was fixed to Δ*E*_0_ ≈ 6.84 eV and ${S}_{0}^{2}\approx 0.92(4)$. From EXAFS fitting by ARTEMIS, atoms were constrained to move into cut-off distances W-O ≈ 1.4–2.8 Å, O-O ≈ 2.2–3.3 Å, W-W ≈ 2.8–4.2 Å. This avoids the atoms getting too close, and the breaking of W-O bonds. Average coordination constraints were set and their weighting was gradually reduced at each RMC-EXAFS cycle. This leads to mean coordination constraints *N*_W-O_ ≈ 4–6 and *N*_W-W_ ≈ 4–6, which were found to be the most suitable steady-state conditions to decrease the residual and reach spectral convergence. Hence, we are using structural constraints to avoid the exploration of configurations far from the already energetically and structurally pre-converged structures. RMC-EXAFS structures of *a*WO_3_ were then optimized allowing a 3–5% of atoms to undergo displacements of ≈0.08 Å, at every RMC-EXAFS cycle. Each atomic movement is evaluated according to the degree of consistency ${\Re }^{2}$ between the experimental and the refined spectral-data-points. Thus, if the atomic movement increases the consistency ${\Re }^{2}$ it is accepted. If instead, it lowers the consistency ${\Re }^{2}$ it is accepted with probability $\wp$ = ${e}^{({\Re }_{0}^{2}-{\Re }_{1}^{2})\mathrm{/2}}$. When the experimental and refined data-points are statistically the same then the value of ${\Re }^{2}$ is less than the number of degrees of freedom^38^. Total ${k}^{3}\chi {(k)}_{{\rm{RMC}}}$ and FT${|{k}^{3}\chi (k)|}_{{\rm{RMC}}}$ functions; equal to the averaged single spectrum of each photoabsorbing W atom were re-calculated at each RMC-EXAFS cycle. Reaching of convergence to a minimum residual of ≈1–5 × 10^−3^ was attained by running ≈8 × 10^5^ RMC-EXAFS cycles. After refinements, all twelve RMC-EXAFS optimized structures of *a*WO_3_ attained similar atomic-bonding distributions and displayed small variation in the atomic coordination and bond-angle distributions. This is of course, due to the use of already pre-converged MD trajectories and structural constraints applied in simulations, which force the input structures to achieve similar structural order to fit the EXAFS spectra. Here the reported structural correlations and physical quantities correspond to the average over those twelve RMC-EXAFS optimized structures.

### Hybrid density functional theory [DFT]

Electronic properties of the RMC-EXAFS optimized structures of *a*WO_3_ were studied by *ab*-*initio* hybrid DFT^39^ and used to assess electronic transitions from the W-[2*p*_3/2_] and O-[1*s*] orbitals to unoccupied hybridized W-[5*d*]-O-[2*p*] and single O-[2*p*] CB orbitals. Structural relaxation was done using the PBE^37^ exchange-correlation potential into the electron projector-augmented wave [PAW] method^40^, as implemented in VASP^29^. A maximal force criterion convergence of 0.01 eV/Å was used and an energy cut-off of 700 eV was used to expand the Kohn-Sham orbitals in the plane wave basis set. A 1 × 1 × 1 Monkhorst-Pack mesh^41^ centered at the ${\rm{\Gamma }}$-point was used for *k*-sampling and the non-local range separated screened hybrid functional HSE06 [10% HF; 90% PBE; $\omega$ = 0.2 Å^−1^]^42^ was used.

### *Ab*-*initio* calculations of RMC-XANES spectra

*Ab*-*initio* calculations of the XANES spectra for RMC-EXAFS optimized structures of *a*WO_3_ were carried out in order to assess the electronic transitions associated to unoccupied states in *a*WO_3_. The W-*L*_3_ and O-*K* edge RMC-XANES spectra were calculated self-consistently by *ab*-*initio* FDM, as implemented in the near-edge structure FDMNES code^30^. The full potential FDM method does not approximate the potential’s form, providing a precise description of occupied and unoccupied electronic states. The energy dependent exchange-correlation potentials by Hedin-Lundqvist and Von Barth were used and evaluated using relativistic DFT. Electronic correlations and spin-orbit coupling were approached by Fock-Dirac schemes. Single W-*L*_3_ and O-*K* edge XANES signals were calculated on grids of 7 Å centered at each photoabsorbing W or O atom and then averaged to a total FDM-RMC-XANES function.

## References

[1] Wu, J., Cao, J., Han, W.-Q., Janotti, A. & Kim, H.-C. *Functional metal oxide nanostructures*. 3–358 (Springer-Verlag, New York, USA, 2012).

[2] Neméth K (2012). Efficient simultaneous reverse Monte Carlo modeling of pair-distribution functions and extended x-ray-absorption fine structure spectra of crystalline disordered materials. J. Chem. Phys..

[3] Krayzman V (2009). A combined fit of total scattering and extended X-ray absorption fine structure data for local- structure determination in crystalline materials. J. Appl. Crystallogr..

[4] Massobrio, C., Du, J., Bernasconi, M. & Salmon, P. S. *Molecular dynamics simulations of disordered materials*. 417–419 (Springer, Switzerland, 2015).

[5] Anderson PW (1975). Model for the electronic structure of amorphous semiconductors. Phys. Rev. Lett..

[6] Mott N (1978). Electrons in glass. Rev. Mod. Phys..

[7] Cohen MH, Fritzsche H, Ovshinsky SR (1969). Simple band model for amorphous semiconducting alloys. Phys. Rev. Lett..

[8] Santos L (2015). Electrochemical devices: Structure and morphologic influence of WO_3_ nanoparticles on the electrochromic performance of dual-phase *a*-WO_3_/WO_3_ inkjet printed films. Adv. Electron. Mater..

[9] Dalavi DS (2013). Efficient electrochromic performance of nanoparticulate WO_3_ thin films. J. Mater. Chem. C.

[10] Monk, P. M. S., Mortimer, R. J. & Rosseinky, D. R. *Electrochromism and electrochromic devices*. 125–190 (Cambridge University Press, New York, USA, 2007).

[11] Triana CA, Niklasson GA (2014). Electrochromic properties of Li^+^-intercalated amorphous tungsten (*a*WO_3−*x*_) and titanium (*a*TiO_2−*x*_) oxide thin films. J. Phys: Conf. Series..

[12] de Wijs GA, de Groot RA (1999). Structure and electronic properties of amorphous WO_3_. Phys. Rev. B..

[13] Lugovskaya LA, Aleshina LA, Kalibaeva GM, Fofanov AD (2002). X-ray study and structure simulation of amorphous tungsten oxide. Acta Cryst, B.

[14] Ankudinov AL, Ravel B, Rehr JJ, Conradson SD (1998). Real-space multiple-scattering calculation and interpretation of x-ray-absorption near-edge structure. Phys. Rev. B..

[15] Tucker MG, Keen DA, Dove MT, Goodwin AL, Hui Q (2007). RMCProfile: reverse Monte Carlo for polycrystalline materials. J. Phys.: Condens. Matter..

[16] Newville M (2014). Fundamental of XAFS. Rev. Mineral. Geochem..

[17] Triana CA, Araujo CM, Ahuja R, Niklasson GA, Edvinsson T (2016). Electronic transitions induced by short-range structural order in amorphous TiO_2_. Phys. Rev. B..

[18] Nanba T, Nishiyama Y, Yasui I (1991). Structural study of amorphous WO_3_ thin films prepared by the ion exchange method. J. Mater. Res..

[19] Bets V (1987). Studies of tungsten oxide electrochromic thin films and polycrystals by the EXAFS method. Nucl. Instr. and Meth. A..

[20] Nanba T (1994). Characterization of amorphous tungsten trioxide thin films prepared by rf magnetron sputtering method. J. Non-Crystalline Solids..

[21] Nanba T, Yasui I (1989). X-ray diffraction study of microstructure of amorphous tungsten trioxide films prepared by electron beam vacuum evaporation. J. Solid. State. Chem..

[22] Zeller HR, Beyeler HU (1977). Electrochromism and local order in amorphous WO_3_. Appl. Phys..

[23] Ankele J, Mayer J, Lamparter P, Steeb S (2006). Evaluation of the Structure of Amorphous Tungsten Oxide W_28_O_72_ by the Combination of Electron-, X-Ray- and Neutron-Diffraction (Three-Beam Experiment). Z. Naturforsch..

[24] Triana CA, Niklasson GA (2015). Electrochromism and small-polaron hopping in oxygen deficient and lithium intercalated amorphous tungsten oxide films. J. Appl. Phys..

[25] Migas DB, Shaposhnikov VL, Borisenko VE (2010). Tungsten oxides. II. The metallic nature of Magnéli phases. J. Appl. Phys..

[26] Gerand B, Nowogrocki G, Guenot J, Figlarz M (1979). Structural study of a new hexagonal form of tungsten trioxide. J. Solid State Chem..

[27] Zheng H (2011). Nanostructured Tungsten Oxide - Properties, Synthesis, and Applications. Adv. Funct. Mater..

[28] Ramana CV, Utsunomiya S, Ewing RC, Julien CM, Becker U (2006). Structural stability and phase transitions in WO_3_ thin films. J. Phys. Chem. B..

[29] Kresse G, Furthmuller J (1996). Efficient iterative schemes for *ab initio* total-energy calculations using a plane-wave basis set. Phys. Rev. B.

[30] Bunau O, Joly Y (2009). Self-consistent aspects of x-ray absorption calculations. J. Phys.: Condens. Matter..

[31] Wang F, Valentin CD, Pacchioni G (2011). Electronic and structural properties of WO_3_: A systematic hybrid DFT study. J. Phys. Chem. C..

[32] Abtew TA, Drabold DA (2007). *Ab initio* models of amorphous Si_1−*x*_Ge_*x*_:H. Phys. Rev. B..

[33] Yamazoe S, Hitomi Y, Shishido T, Tanaka T (2008). XAFS study of tungsten *L*_1_- and *L*_3_-edges: Structural analysis of WO_3_ species loaded on TiO_2_ as a catalyst for photo-oxidation of NH_3_. J. Phys. Chem. C..

[34] Purans J, Kuzmin A, Parent P, Laffon C (2001). X-ray absorption study of the electronic structure of tungsten and molybdenum oxides on the O *K*-edge. Electrochem. Acta.

[35] Carlson S, Clausen M, Gridneva L, Sommarin B, Svensson C (2006). XAFS experiments at beamline I811, MAX-lab synchrotron source, Sweden. J. Synchrotron. Rad..

[36] Ravel B, Newville M (2005). ATHENA, ARTEMIS, HEPHAESTUS: data analysis for X-ray absorption spectroscopy using IFEFFIT. J. Synchrotron. Rad..

[37] Perdew JP, Burke K, Ernzerhof M (1996). Generalized gradient approximation made simple. Phys. Rev. Lett..

[38] McGreevy RL, Pusztai L (1988). Reverse Monte Carlo Simulation: A New Technique for the Determination of Disordered Structures. Molecular Simulation..

[39] Kohn W, Sham LJ (1965). Self-consistent equations including exchange and correlation effects. Phys. Rev..

[40] Bloch PE (1994). Projector augmented-wave method. Phys. Rev. B..

[41] Monkhorst HJ, Pack JD (1976). Special points for Brillouin-zone integrations. Phys. Rev. B..

[42] Paier J (2006). Screened hybrid density functionals applied to solids. J. Chem. Phys..

